# Fermentation Products of *Paenibacillus bovis* sp. nov. BD3526 Alleviates the Symptoms of Type 2 Diabetes Mellitus in GK Rats

**DOI:** 10.3389/fmicb.2018.03292

**Published:** 2019-01-09

**Authors:** Zhenyi Qiao, Jin Han, Huafeng Feng, Huajun Zheng, Jiang Wu, Caixia Gao, Meng Yang, Chunping You, Zhenmin Liu, Zhengjun Wu

**Affiliations:** ^1^State Key Laboratory of Dairy Biotechnology, Shanghai Engineering Research Center of Dairy Biotechnology, Dairy Research Institute, Bright Dairy & Food Co., Ltd., Shanghai, China; ^2^College of Food Science and Technology, Shanghai Ocean University, Shanghai, China; ^3^Shanghai-MOST Key Laboratory of Health and Disease Genomics, Chinese National Human Genome Center at Shanghai, Shanghai, China; ^4^Key Laboratory of Reproduction Regulation of NPFPC, Shanghai Institute of Planned Parenthood Research, IRD, Fudan University, Shanghai, China; ^5^School of Life Sciences, Shanghai University, Shanghai, China

**Keywords:** type 2 diabetes, gut microbiota, *Paenibacillus bovis* sp. nov. BD3526, *Akkermansia muciniphila*, cytokine

## Abstract

Gut microbiota is closely related to type 2 diabetes mellitus (T2DM). The gut microbiota of patients with T2DM is significantly different from that of healthy subjects in terms of bacterial composition and diversity. Here, we used the fermentation products of *Paenibacillus bovis* sp. nov. BD3526 to study the disease progression of T2DM in Goto-kakisaki (GK) rats. We found that the symptoms in GK rats fed the fermentation products of BD3526 were significantly improved. The 16S rRNA sequencing showed that the fermentation products of BD3526 had strong effects on the gut microbiota by increasing the content of *Akkermansia*. In addition, the interaction of the genus in the gut of the BD3526 group also significantly changed. Additional cytokine detection revealed that the fermentation products of BD3526 can reduce the inflammatory factors in the intestinal mucus of GK rats and thereby inhibit the inflammatory response and ameliorate the symptoms of T2DM.

## Introduction

According to data published by the International Diabetes Federation, there were 425 million people with diabetes worldwide in 2017. Type 2 diabetes mellitus (T2DM) is characterized by a sustained decrease in the insulin secretion of pancreatic β-cells, which leads to insufficient insulin to fulfill the requirement of the body. Long-term T2DM in the human body can cause serious complications, for example in the kinds of kidney disease, cancer and cardiovascular disease (Stern, 1995; Coughlin et al., 2004).

In the last two decades, evidence has accumulated that the pathogenesis of T2DM and its complications may be related to inflammation factors (i.e., IL-1β, IL-6, MCP-1, TNF-α and IFN-γ) (Cuman et al., 2001; Rotter et al., 2002; Masters et al., 2010; Westwellroper et al., 2011; WestwellRoper et al., 2014; Greer et al., 2016). For example, IL-6 is the most endocrine cytokine and is not only produced by immunocompetent cells but also by fat cells involved in the body’s inflammatory response and energy metabolism (Rotter et al., 2002). In patients with inflammation, the IL-6 levels are elevated. Excess IL-6 promotes pancreatic islet β lymphocyte differentiation and overexpresses the IgG gene, which promotes excessive activation of T lymphocytes and thereby causes destruction and death of islet β cells (André-Schmutz et al., 2010; Donath, 2013). Therefore, the therapeutic targets for T2DM have shifted from simple hypoglycemic drugs (e.g., insulin and acarbose) to drugs suppressing inflammatory factors (such as IL-1 receptors blockade Anakinra and IKKβ-NF-κB inhibition Salsalate) (Donath, 2013).

The gut microbiota plays an essential role in the development of T2DM by skewing the host immune response to the inflammatory reaction (Karlsson et al., 2013). Wu et al. (2017) found that clinical patients with T2DM had significant changes in the composition and diversity of the gut microbiota after taking metformin being beneficial to improve the symptoms of T2DM. Some sources claim that *Akkermansia muciniphila* in the feces is significantly enriched in patients and mice with T2DM after taking metformin (Karlsson et al., 2013; Lee and Ko, 2014; Shin et al., 2014; Zhang et al., 2015; de la Cuesta-Zuluaga et al., 2017; Forslund et al., 2017). Low levels of *A*. *muciniphila* in the intestine may correlate with the thinning of the mucosal layer, which leads to a weakened intestinal barrier function (Derrien et al., 2004). In addition, *A. muciniphila* was reduced in patients with obesity and T2DM (Derrien et al., 2004; Everard et al., 2013). Further out, a recent study also claims that *A*. *muciniphila* can improve the response rate of tumor patients to PD-1/PD-L1 immunotherapy (Routy et al., 2018).

We have since isolated a novel bacterium designated as *Paenibacillus bovis* sp. nov. BD3526 from Tibetan yak milk in a previous work (Hang et al., 2016). This strain can synthesize exopolysaccharides with immunomodulatory activity *in vitro* (Xu et al., 2016). Instead, attempts to identify the relationship between exopolysaccharides and T2DM are not yet resolved. In this work, the lyophilized supernatant of the fermented milk by the BD3526 strain was fed to GK rats and its effect on the diabetic phenotype of the rats was observed. It was found that the symptoms of diabetes in the rats fed the supernatant were significantly improved. The content of *A*. *muciniphila* in the intestines was significantly increased, which coincided with a decrease in the inflammatory response in the intestine.

## Materials and Methods

### Bacterial Strain and Cultivation

*Paenibacillus bovis* sp. nov BD3526 (CGMCC 8333 = DSM28815 = ATCC BAA-2746) was provided by the State Key Laboratory of Dairy Biotechnology, Shanghai 200436, China. The bacterial strain was routinely cultivated aerobically on milk agar at 30°C for 24 h. The medium was prepared by adding 10 mL sterile 10% (w/w) reconstituted skim milk to 100 mL melted 1.5% (v/w) agar solution. The strain was stored in sterile 10% (w/w) reconstituted skim milk supplement with 10% (v/v) glycerol at -80°C.

### Preparation of the BD3526 Strain Fermentation Products

A loop of freshly cultivated BD3526 on milk agar was inoculated into a 100-mL flask containing 20 mL sterile 10% (w/w) reconstituted skim milk and cultivated at 30°C at 180 rpm for 24 h. The culture was then transferred to a 250-mL flask containing 50 mL sterile 10% (w/w) reconstituted milk at a ratio of 4% (v/v) and cultivated at the same conditions as mentioned above for 72 h. Samples at different intervals were boiled for 5 min and then centrifuged at 12000 ×*g* at 4°C for 20 min. The supernatant was neutralized with 1 M NaOH to pH 6.8 and assayed for the inhibitory activity to alpha glucosidase (EC 3.2.1.20) utilizing *p*-nitrophenyl a-D-glucopyranoside (PNPG) as the substrate. The 72 h culture with an inhibitory activity to alpha glucosidase of approximately 60% (data unpublished) was boiled and centrifuged at the same condition mentioned above. The supernatant was lyophilized under a vacuum. The lyophilized powder was then cold stored. Before gavage of the experimental animals, the powder was redissolved in distilled water at a concentration of 50 mg/mL.

### Animal Experiments

A total of 10 twelve-week-old Goto-kakisaki (GK) rats were adopted for 1 week and then randomly divided into two groups. The rats in the BD3526 group were gavaged daily with 2 mL 50 mg/mL lyophilized fermentation products of BD3526, whereas the rats in the control group were gavaged with 2 ml 50 mg/mL skim milk powder. The animals were individually caged with free access to a normal chewing bar and drinking water. On the day of the assay of postprandial blood glucose, the animals were first fed a normal chewing bar for 1 h and then gavage was conducted. After the gavage, the chewing bars were removed from the cage and a blood sample from the tail was collected 2 h after the gavage and analyzed with commercial blood glucose test strips (Sannuo, Shenzhen, China).

The postprandial blood glucose and body weight were measured weekly and fecal samples were collected. Glycated hemoglobin was measured at week 5. From the 6th week and thereafter, the gavage was terminated and the animals were restored to normal management. Postprandial blood glucose and body weight was continuously measured (weekly) and fecal samples were collected until the 9th week. After the 9th week, the intestinal mucus of the rats was collected for detection of the immunological factor. Feeding of the rates was conducted at Shanghai Slac Experimental Animal Co., Ltd.

### DNA Extraction and PCR Amplification

Microbial DNA was extracted from the fecal samples using the E.Z.N.A.^®^ stool DNA Kit (Omega Biotek, Norcross, GA, United States), according to the manufacturer’s protocols. The final DNA concentration and purification were determined by a NanoDrop 2000 UV-vis spectrophotometer (Thermo Scientific, Wilmington, DE, United States), and the DNA quality was checked by 1% agarose gel electrophoresis. The V3-V4 hypervariable regions of the bacteria 16S rRNA gene were amplified by a thermocycler PCR system (GeneAmp 9700, ABI, United States). The PCRs were performed in triplicate with the 20-μL mixture containing 4 μL 5 × FastPfu Buffer, 2 μL 2.5 mM dNTPs, 0.8 μL of each primer (5 μM), 0.4 μL FastPfu Polymerase and 10 ng template DNA. The resulting PCR products were extracted from a 2% agarose gel, further purified using the AxyPrep DNA Gel Extraction Kit (Axygen Biosciences, Union City, CA, United States) and quantified using QuantiFluor TM-ST (Promega, United States), according to the manufacturer’s protocol.

### High-Throughput Sequencing

The purified amplicons were pooled in equimolar and paired-end sequenced (2 × 300 bp) on an Illumina MiSeq platform (Illumina, San Diego, CA, United States), according to the standard protocols by Sinotech Genome Technology Co., Ltd. (Shanghai, China).

### Bioinformatic Analysis and Statistical Analysis

In the bioinformatics analysis of 16S rRNA sequencing samples, we used an online cloud platform from Sinotech Genome Technology Co., Ltd.

Specifically, we used Usearch (version 7.0)^1^ to perform cluster analysis on the OTUs. The diversity of the BD3526 group and the control group was analyzed using mother software (version v.1.30.1)^2^. Furthermore, we also used the PLS-DA analysis in the R language mixOmics package and created a distance map for each sample.

In the comparison of different strains, we used metagenome seq derived from the R language package to perform the Zero-inflated Gaussian distribution to process the impact of sequencing depth, and we finally found a difference based on the linear model.

In the random forest analysis, we used the Random Forest package and the plotROC package to quickly and efficiently select the species category that is most important for sample classification and to perform relevant ROC verification.

We used Networkx software^3^ in the collinear network analysis and correlation network analysis.

The 16S function prediction is used to standardize the OTU abundance table by PICRUSt (the PICRUSt software stores the KO information corresponding to the greengene id), that is, to remove the influence of the number of copies of the 16S marker gene in the species genome the. We then pass the greengene id corresponding to each out, obtain the KEGG Ortholog (KO) information corresponding to the out, and calculate the KO abundance. According to the KEGG database information, the KO, Pathway, and EC information can be obtained, and the abundance of each functional category can be calculated based on OTU abundance. In addition, for the Pathway, PICRUSt can be used to obtain three levels of information on the metabolic pathways and to obtain abundance tables for each level.

In addition, we also used Graphpad Prism6 software to create statistical images of the statistical data.

### *In vitro* Gut Simulator

The basic protocols were referred to the published article (Wu et al., 2017). Briefly, 2% fecal samples were gathered and cultured in 5 mL BHI broth for 3 h at 37°C and the 2% preculture was seeded into the feed medium. The medium was composed by: (in g/liter) arabinogalactan (1.0), pectin (2.0), xylose (1.5), starch (3.0), glucose (0.4), yeast extract (3.0), peptone (1.0), mucin type II (4.0), and cysteine (0.5). The medium was acidified to around pH 2 with 6-M HCl to simulate digestion processes, and neutralized with simulated pancreatic juice to a pH of around 6.9. The simulated pancreatic juice contained: (in g/liter) NaHCO_3_ (12.5), Oxgall bile salts (6.0), and pancreatin (0.9). Unless the experiment performed for 3 days, all the cultures were under the condition of anaerobic.

### Cytokine Detection

The protocol of cytokine detection can be referred to from the instructions of a rat Cytokine Antibody Array Kit (Abcam, ab133992). Briefly, the membrane was incubated with blocking buffer. Then, 1 mL large intestine mucus was added to bind the antibodies that are fixed at the surface of the membrane. Following this step, the membrane was washed with washing buffer five times and then incubated with 1X biotin-conjugated anti-cytokines and 1X HRP-conjugated streptavidin. Finally, the membrane was exposed by X-ray film.

### Accession Numbers

The *Paenibacillus bovis* sp. nov. BD3526 mentioned in this article can be found in the ATCC database. The ATCC number of *Paenibacillus bovis* sp. nov. BD3526 is BAA-2746. High-throughput sequencing data from this study was deposited in the Sequence Read Archive (SRA) databases under the following accession number: SRP151163 and PRJNA508215.

### Ethics Statement

The project use and care of the animals in this research was reviewed and approved by the Shanghai Laboratory Animal Management Office (SYXK [Shanghai] 2017-0008).

The animals used in the research were utilized based on appropriate experimental procedures. All of the animals were lawfully acquired, and their retention and use were in compliance with federal, state and local laws and regulations in every case and in accordance with the Institutional Animal Care and Use Committee of SLAC (IACUC) Guide for Care and Use of Laboratory Animals.

Animals used in this research received every consideration for their comfort and were properly housed, fed, and their surroundings kept in a sanitary condition.

The use of animals was in accordance with the IACUC Guide for Care and Use of Laboratory Animals. A minimal number of mice were used during the experiments. Appropriate anesthetics were used to eliminate sensibility to pain during all of the surgical procedures.

## Results

### Diabetic Symptoms Were Alleviated in the BD3526 Group

**FIGURE 1:**
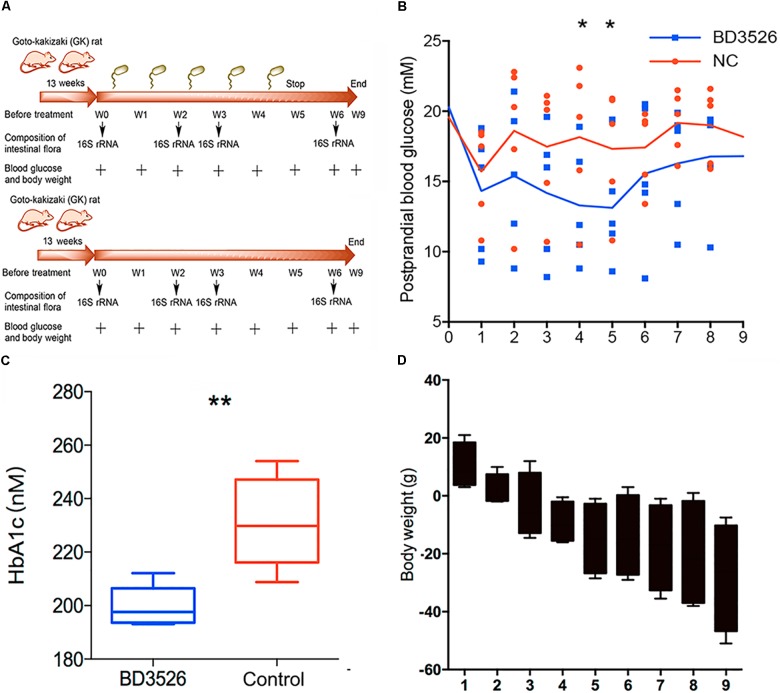
Observation of type 2 diabetes mellitus symptoms. **(A)** A total of 10, 13-week-old GK rats were used in this work and randomly divided into two groups. The rats in the BD3526 group were gavaged daily with 2 mLlyophilized powder of the BD3526 strain fermentation products (50 mg/mL), whereas the GK rats in the control group were gavaged daily with 2 mL physiological saline. All of the animals were permitted free access to normal chewing bars and water. At intervals of 0, 2, 3, and 6 weeks, the changes of diabetes indexes in these rats were observed. At the sixth week, the gavage of either BD3526 strain fermentation products or physiological saline was interrupted and the rats were restored to normal diets. After the restoration, the animals were anesthetized and killed, and the mucus in the intestine and colon was scratched. It is desirable to observe changes in the phenotype after stopping the ingestion of the BD3526 strain fermentation products. **(B)** Postprandial blood glucose measurements were performed in the BD3526 group and the control group. The *x*-axis represents the time of the week. The *y*-axis represents the postprandial blood glucose concentration (mM/L). The asterisk represents a significant difference in blood glucose concentration between the BD3526 group and the control group (^∗^*P*-value < 0.05, mean ± SEM). **(C)** The glycated hemoglobin (HbA1c) test confirmed that the blood glucose concentration in the BD3526 group was significantly lower than that in the control group (^∗∗^*P*-value < 0.01, mean ± SEM). **(D)** Body weight indicators were used to assess weekly body weight changes in the BD3526 group and control group models (mean ± SEM).

In our previous work, we found that the BD3526 strain could synthesize a large amount of exopolysaccharides (36.25 g/L) with *in vitro* immunomodulatory activity (Xu et al., 2016), which might also play roles in retarding the development of diabetes *in vivo*. Besides EPS, monosaccharides, for example in the shapes of fructose, could also be identified in the fermentation products of BD3526. Therefore, to observe the effect of the BD3526 strain fermentation products in skim milk on blood glucose, we selected ten GK rats as subjects. GK rats are a commonly used model of spontaneous nonobese type 2 diabetes with mildly elevated fasting blood glucose, elevated blood glucose after eating, and stable glucose-stimulated insulin secretion disorders and glucose intolerance (Figure 1A). In addition, it has similar changes to human microvascular complications of type 2 diabetes. Its phenotypes are approaching stability when closing to adulthood. The results demonstrated that postprandial blood glucose in the BD3526 group showed a significant decrease in the fourth and fifth week (^∗^*P*-value < 0.05, mean ± SEM) (Figure 1B). The lowest point of postprandial blood glucose in the BD3526 group appeared in the fifth week, and the blood glucose concentration was 13.12 mM/L. Correspondingly, the postprandial blood glucose concentration of the control group of GK rats was 17.32 mM/L in the fifth week. In addition, the glycosylated hemoglobin index of the BD3526 group was also significantly lower than that of the control group (^∗∗^*P*-value < 0.01, mean ± SEM) (Figure 1C). The average concentration of glycated hemoglobin in the BD3526 group was 199.58 nM/L, whereas the value in the control group was 231.25 nM/L. This finding suggests that the diabetic symptoms of the BD3526 group were significantly alleviated. Furthermore, in the body mass index test, we subtracted the body weight of the control group rats from that of the BD3526 group and observed that the weight gain rate of GK rats in the BD3526 group was significantly lower than that in the control group (Figure 1D). These results indicate that the BD3526 strain fermentation products had the ability to reduce diabetes-related indicators in GK rats of T2DM.

### The Diversity of Gut Microbes in GK Rats Fed With the Fermentation Products of the BD3526 Strain Increased

In addition to weekly testing of physiological and biochemical indicators of GK rats in the BD3526 group and the control group, fecal samples were also collected at weeks 0, 2, 3, and 6 for 16S rRNA sequencing. It is hoped that the effect on the gut microbes of GK rats can be observed. Among them, week zero and the sixth week were the standards of the initial and washout value of the GK rat fecal microbiota in the BD3526 group, respectively, and the second week and the third week represented the microbiota affected by the intake of the BD3526 fermentation products.

**FIGURE 2:**
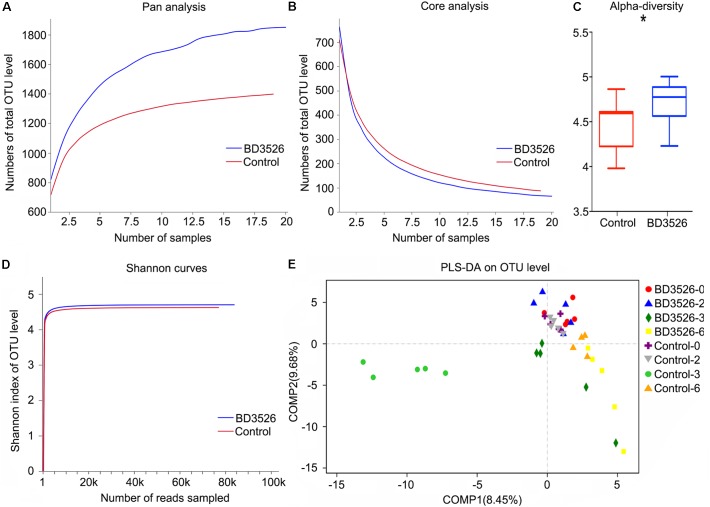
Analysis of the gut microbiota diversity. **(A)** A pan analysis was used to observing the increase in the total number of species as the number of samples increases. **(B)** A core analysis was used to observe the decrease in the number of common species as the number of samples increased. **(C)** The α-diversity statistics show the diversity index values of each sample under each index type. (^∗^*P*-value < 0.05, mean ± SEM). **(D)** The 97% similarity OTU or other taxonomic levels were selected to use mothur software to calculate the diversity index under different random sampling and to use the R-package tools to create the Shannon curve. **(E)** The PLS-DA algorithm distinguishes sample groups of different time periods in the BD3526 group and the control group.

The microbiota in the fecal samples of either the BD3526 group or the control were assayed by 16S rRNA sequencing performed on the Illumina Miseq platform. After sequencing, the biodiversity of the gut microbes of the two groups of GK rats were analyzed. At weeks 2 and 3, Pan/Core analysis showed that in the BD3526 group, the total number of OTUs was significantly higher than that in the control group (Figure 2A). Correspondingly, the number of OTUs shared in both the control group and the BD3526 group showed a significant decrease (Figure 2B). The Shannon curve also showed that the number of OTUs in the BD3526 group and that in the control group were saturated with the increase in sequencing reads, and the number of OTUs in the BD3526 group was greater than that in the control group (Figure 2D). These results indicate that the gut microbiota diversity of GK rats in the BD3526 group was significantly higher than that in control GK rats. This result was also confirmed by alpha diversity verification (^∗^*P* < 0.05, mean ± SEM) (Figure 2C).

To perform a difference analysis within and between groups, a PLS-DA (Partial Least Squares Discriminant Analysis) algorithm was chosen to compare the two groups of GK for week 0, week 2, week 3, and week 6. As shown in Figure 2E, no significant difference between the BD3526 group and the control group was observed. Instead, with the administration of the BD3526 fermentation products, the gut microbiota was diversified, and at the third week, the difference between the BD3526 group and the control group was the most significant. Nevertheless, at the 6th week and after termination of administration of the BD3526 fermentation products for 1 week, the difference in the gut microbiota between the two groups was partially restored.

### *Akkermansia* Is Greatly Enriched in the Gut of GK Rats Fed BD3526 Fermentation Products

**FIGURE 3:**
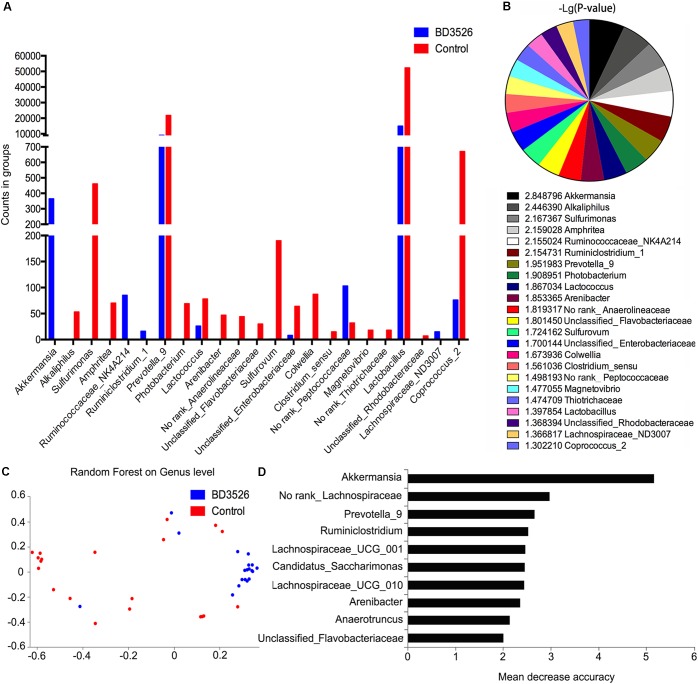
Screening of the different genera. **(A)** A metagenomeseq method was used to screen different strains of the BD3526 group and control group. The blue columns represent the enrichment of different strains in the BD3526 group. The red columns represent the enrichment of different strains in the control group. **(B)** *P*-value distribution of differential strains screened by metagenomeseq method. **(C)** Two-dimensional scatter plot prediction of BD3526 and control samples by random forest distribution. **(D)** Rapid selection of species categories that are most important for sample classification through random forest distribution. The highest mean decrease accuracy of the genus represented the greatest impact on the BD3526 and control components.

The 16S rRNA sequencing is mainly concerned with differences in gut microbiota composition between groups. Therefore, we used the metagenomeseq algorithm to analyze the differences in gut microbiota composition between BD3526 GK rats and control GK rats at weeks 2 and 3. Based on the statistics of the counts of each group of sequencing samples, we found that a total of 23 genera changed in the BD3526 group (*P* < 0.05). Among them, *Akkermansia*, *Ruminococcaceae*_NK4A214, *Ruminclostridum*_1 and No rank_*Peptococcaceae* were elevated in the BD3526 group (Figure 3A and Supplementary Table S1). A statistical analysis of the FDR-corrected *P*-values of these 23 genera revealed that *Akkermansia* demonstrated the most significant difference among all of the changed genera [-10 Lg(*P*-value) = 2.848796] (Figure 3B). In the BD3526 group, *Akkermansia*, *Ruminococcaceae*_NK4A214, *Ruminiclostridium*_1 and *Lachnospiraceae*_ND3007 were enriched, while the four genera were not detected in the other group. Instead, *Alkaliphilus*, *Sulfurimonas*, *Amphritea, Photobacterium*, *Arenibacter*, *Anaerolineaceae*, *Flavobacteriaceae*, *Sulfurovum*, *Colwellia*, *Clostridium sensu*, *Magnetovibrio*, *Thiotrichaceae,* and *Rhodobacteraceae* disappeared in the BD3526 group. To be precise, after correcting *Akkermansia* to species, we found that it corresponds to *A. muciniphila*.

However, the metagenomeseq algorithm itself has certain limitations. To eliminate the ostensible impact of gut microbiota diversity caused by the genus due to its low abundance or low differential fold and efficiently identify the most important OTUs for the BD3526 group, we also constructed a model of random forest distribution (Figure 3C). In this model, the gut microbiota in the BD3526 group and the control group were enriched in two different sites. We found that *Akkermansia* had the most important position in this model based on the genus abundance of random forest distribution (Figure 3D and Supplementary Table S2). This suggests that *Akkermansia* may be the biggest influencing factor for the difference between the BD3526 group and the control group. In addition to *Akkermansia*, No rank_*Lachnospiraceae*, *Prevotella*_9, *Ruminiclostridium*, *Lachnospiraceae*_UCG_001 and *Candidatus_Saccharimonas* were also among the top five genera. Among them, *Akkermansia* and *Prevotella*_9 were also the two genera screened by the metagenomeseq algorithm that showed a significant difference between the two rat groups.

In the random forest distribution analysis, we also used the top 6 genera of the mean reduction accuracy (*Akkermansia*, No rank_*Lachnospiraceae*, *Prevotella*_9, *Ruminiclostridium*, *Lachnospiraceae*_UCG_001 and *Candidatus_Saccharimonas*) as models to perform the receiver operating characteristic curve analysis (ROC curve) (Supplementary Figure S1). The results show that in the model containing only these six genera, the Receiver Operating Characteristic Curve (ROC) reached 0.81. Correspondingly, in the model containing all genera, the ROC is 0.48. When the six genera were removed from the model of all genera, the ROC was 0.51. This illustrated that our model was accurate to assess the difference between the two groups.

Interestingly, among the six genera, *Akkermansia* displayed the most significant difference in 16S rRNA sequencing, which suggests that *Akkermansia* may play an important role in ameliorating the symptoms of the GK rats fed BD3526 fermentation products. Although short-chain fatty acids (SCFAs) had been reported as key effectors in T2DM by other researchers (Zhao et al., 2018), no enrichment of SCFAs-producing microbiota was observed in the BD3526 group.

For further confirm the direct relationship between fermentation products and *Akkermansia*, we re-cultured the fecal samples *in vitro* which treated with 5% fermentation products or 5% skim milk. In this *in vitro* experiment, we focused *Akkermansia* was significantly enriched after treated with fermentation products for 1 day (Supplementary Figure S2). This phenomenon was consistent to the conclusions we claimed before that *Akkermansia* is greatly enriched in the gut of GK rats fed BD3526 fermentation products.

### The BD3526 Strain Fermentation Products Shifted the Interactions of the Gut Microbes

**FIGURE 4:**
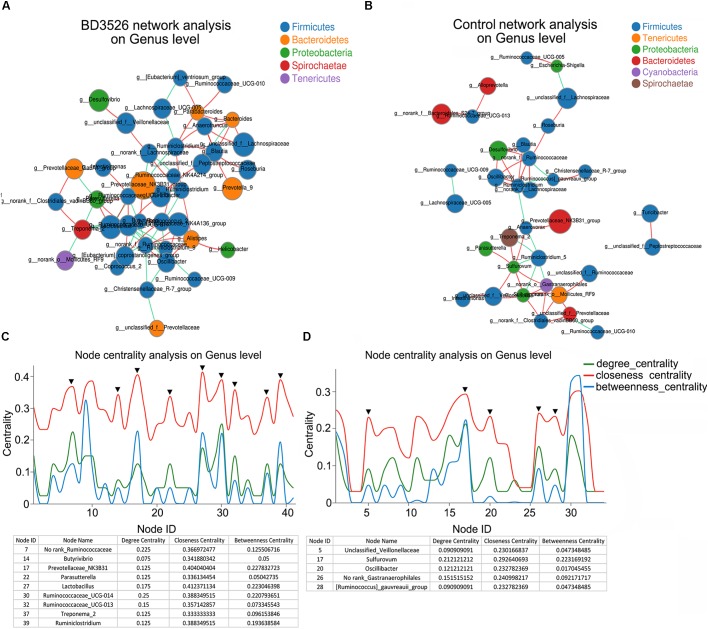
Interaction network of gut microbiota. **(A)** The dominant microbiota interaction network in the BD3526 group. **(B)** The dominant microbiota interaction network in the control group. **(C)** The key strain nodes in the BD3526 group were analyzed for the core genus. The green curve represents the degree centrality. The red curve represents the closeness centrality and the blue curve represents the betweenness centrality. The nodes whose three curves reach the peak at the same time may be the genus that plays an important role in the interaction network. The detailed genus name and parameter information are listed in the following table. The black triangles represent the important genus in this network. **(D)** The key strain nodes in the BD3526 group were analyzed for the core genus. The green curve represents the degree centrality. The red curve represents the closeness centrality and the blue curve represents the betweenness centrality. The nodes whose three curves reach the peak at the same time may be the genus that plays an important role in the interaction network. Detailed genus name and parameter information are listed in the following table. The black triangles represent the important genus in this network.

In the gut, microbe interactions between different genera and between different species often occurred. Therefore, to further evaluated the effect of the BD3526 strain fermentation products on the gut microbiota of GK rats, we used Networkx software for interaction network analysis. The interaction between microorganisms of different genera in the same sample and species correlation between different samples were evaluated. In the species interaction network of the BD3526 group and the control group, we found that the dominant species belong to Firmicutes. At the phylum level, there was no significant difference between the two groups (Figures 4A,B). However, when we constructed the node centrality diagram at the genus level, we found that the interaction network of the BD3526 group and the control group were significantly different. In the node centrality diagram, the number on the *x*-axis represents a different node. Each node corresponds to the interaction of the individual genus to the other genera. When a peak simultaneously occurred on the three curves of the degree centrality, closeness centrality and between centrality, it was considered that the corresponding genus of the node might be of great significance to the entire network. According to this principle, 9 genera were identified in the BD3526 group including No rank_*Ruminococcaceae*, *Butyrivibrio*, *Prevotellaceae*_NK3B31, *Parasutterella* and *Lactobacillus*, *Ruminococcaceae*_UCG-014, *Ruminococcaceae*_UCG-013, *Treponema*_2 and *Ruminiclostridium*. In the control group, only five genera were identified including Unclassified_*Veillonellaceae*, *Sulfurovum*, *Oscillibacter*, No rank_*Gastranaerophilales*, and [*Ruminococcus*]_*gauvreauii*_group (Figures 4C,D). There were no associations between the 9 genera of the BD3526 group and the 5 genera of the control group. This result suggests that the fermentation products of the strain BD3526 may exert an important impact on the gut microbiota interaction network of GK rats. This may be another important factor to improve the symptoms of T2DM in the BD3526 group.

At the same time, the species correlation revealed that the rats in the BD3526 strain group shared a portion of the species with those of the control group. However, the points corresponding to the BD3526 group alone were significantly higher than those of the control group alone (Supplementary Figure S3), which indicated that the species in the gut microbiota of GK rats in the BD3526 group demonstrated more specificity.

### The BD3526 Strain Fermentation Products Decreased the Genera in the Gut Microbiota That Were Closely Related to Diseases in GK Rats

**FIGURE 5:**
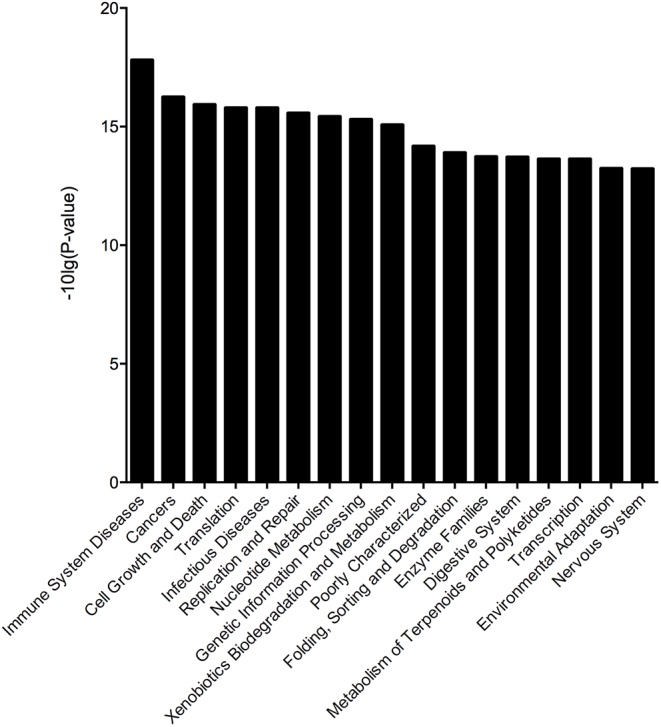
Microbial changes in the BD3526 group. The *x*-axis represents the KO name of the signal pathways. The *y*-axis represents the difference in gene annotation of the KEGG (Pfdr < 0.05) between the BD3526 group and the control group.

To assess the effect of different microbes on the stability of the gut microbiota in the BD3526 group, we obtained 16S rRNA sequencing data through the corresponding Greengene ID of each OTU. The functional composition profiles were predicted with PICRUSt. The results show that in the BD3526 group, the most significant changes in KEGG (level 2) categories were focused on five pathways including immune system diseases [-10 Lg (*P*-value) = 17.798790], cancer [-10 Lg (*P*-value) = 16.241620], cell growth and death [-10 Lg (*P*-value) = 15.919890], translation [-10 Lg (*P*-value) = 16.241620] and infectious diseases [-10 Lg (*P*-value) = 15.785920] (Figure 5). These five KEGG pathways were all associated with diseases including immune disease, cancer and infectious diseases. This suggests that the intake of the BD3526 strain fermentation products may have a profound effect on the development of diseases and maintain the balance of the gut microbiota of GK rats.

### The BD3526 Strain Fermentation Products Lowered the Intestinal Mucosal Inflammation Response in GK Rats

The causes of T2DM are often complicated. Studies have reported that the onset of T2DM was correlated with a chronically low-grade inflammatory response (Donath et al., 2003; Ehses et al., 2009; Masters et al., 2010; Westwellroper et al., 2011; Lerner et al., 2012; Oslowski et al., 2012; Jourdan et al., 2013; WestwellRoper et al., 2014). The inflammatory response plays an important role in the occurrence and development of diabetes. Another study reported that *A. muciniphila* can increase intestinal mucus thickness and reduce the inflammation response (Wu et al., 2017).

**FIGURE 6:**
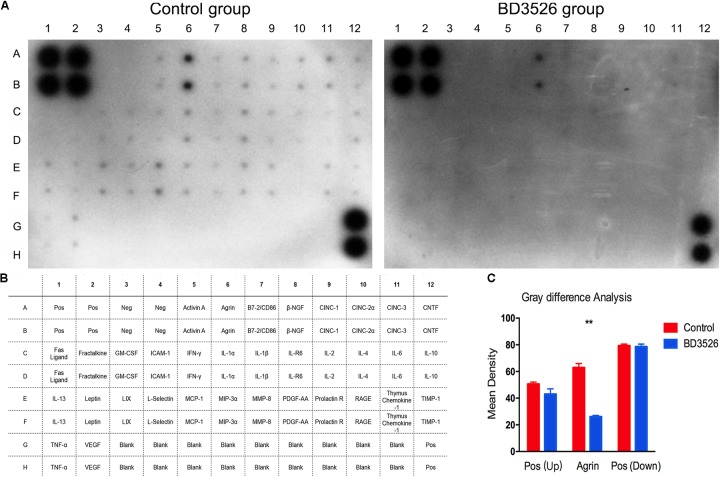
Intestinal mucosal cytokines detection. **(A)** The cytokines of the control group and the BD3526 group were both detected. **(B)** In the membrane, all of the spots were listed in the table below. In addition to all 34 cytokines, A1, A2, B1, B2, G12, and H12 were positive controls. G3 to G11 and H3 to H11 were all negative controls. **(C)** Cytokines were semi-quantitatively analyzed using ImageJ software (^∗∗^*P*-value < 0.01, mean ± SEM).

As aforementioned, we found that the BD3526 strain fermentation products significantly increased *A. muciniphila* in GK rats and reduced the genera in the gut microbiota closely related to diseases by the KEGG analysis. Therefore, we postulated that the BD3526 strain fermentation products could reduce the inflammatory factors of intestinal mucosal by increasing the *A. muciniphila* content and ultimately play a role in lowering blood glucose. Assuming this postulation, we extended our work to the inflammatory factors in the intestinal mucosa of the BD3526 group and the control group by using a rat Cytokine Antibody Array Kit (Abcam, ab133992). After a period of 5 weeks of washout, the cytokines expressed in the large intestinal mucus of the rats in the BD3526 group and those in the control group were assayed. The kit can simultaneously detect 34 cytokines in one array. In the large intestinal mucus of the rats in the control group, 32 cytokines, including inflammatory factors (IL-1β, IL-6, MCP-1 and TNF-α) and other oncogenic factors, were expressed at a high level in the GK rats, whereas the Neg and Fas ligand could hardly be detected (Figures 6A,B). Among the 32 cytokines increasingly expressed, an oncogenic factor Agrin (A6 and B6 spots in the array map) was remarkably enriched in GK rats. In the other 31 cytokines, IL-1β (C7 and D7 spots in the array map) is regarded as a cytokine that activates multiple immune cells and promotes insulin resistance (Dinarello, 2009). IFN-γ (C5 and D5 spots in the array map) is reported to be a mediator in the regulation of glucose metabolism by *A. muciniphila* (Greer et al., 2016). IL-6 (C11 and D11 spots in the array map) is confirmed to be a promoter of the death of islet β cells, which leads to T2DM (Donath, 2013). Correspondingly, the expression of cytokines in the BD3526 group was generally decreased (Figures 6A,B). To further semi-quantify the expression of inflammatory factors, we used ImageJ software to perform gray-scale analysis on the two images. The results showed that the intensity of the positive controls in the upper left and lower right are similar in the two arrays. However, the only expressed gene Agrin in the BD3526 group decreased by approximately 50% compared with the control group (Figure 6C). This result implied that the BD3526 strain fermentation products could reduce the expression level of most cytokines in the large intestinal mucus, such as IL-1β, IFN-γ and IL-6, and thus lower the blood glucose in GK rats.

## Discussion

The gut microbiota is widely involved in the development of various diseases, and alteration of the gut microbiota through the intake of some specific microbial supplements has been adopted for the treatment of various diseases. These diseases include autism, Parkinson’s disease and cancer (Hsiao et al., 2013; Buffington et al., 2016; Thaiss et al., 2016; Yu et al., 2017; Chung et al., 2018; Dejea et al., 2018; Routy et al., 2018; Tilg et al., 2018). In the study of gut microbiota and diabetes, the diversity and composition of the gut microbiota of patients changed significantly compared with healthy people (Cani et al., 2008; Larsen et al., 2010; Musso et al., 2010; Qin et al., 2012). In several intervention experiments, metformin has been found to increase the content of *A. muciniphila* and other short-chain fatty acid-producing microorganisms in the intestine of diabetic patients (Shin et al., 2014; de la Cuesta-Zuluaga et al., 2017; Wu et al., 2017; Wang et al., 2018). Metformin also has a profound effect on the interaction of microorganisms within the gut microbiota. In this work, when GK rats were administered with the BD3526 strain fermentation products, the content of *A. muciniphila* in the intestine increased significantly compared with those in the control group. Similarly, *A. muciniphila* showed an increase after the metformin treatment in T2DM subjects (Shin et al., 2014; de la Cuesta-Zuluaga et al., 2017). This outcome indicates that metformin and the BD3526 strain fermentation products might behave similarly in alleviating the symptoms of diabetes. However, administration of metformin not only increase the content of *A. muciniphila* but also the content of other SCFAs-producing microorganisms in the intestine, e.g., *Butyrivibrio*, *Bifidobacterium bifidum*, and *Megasphaera* (de la Cuesta-Zuluaga et al., 2017), which provide a rational explanation for the higher levels of SCFAs being detected in the stool of clinical patients taking metformin (Wu et al., 2017). A large amount of dietary fiber has also been demonstrated to promote the growth of some probiotics in the intestine, stimulate the production of SCFAs, and alleviate the symptoms of diabetes (Zhao et al., 2018). Nevertheless, GK rats fed the BD3526 strain fermentation products did not exhibit a similar phenomenon, i.e., SCFAs-producing microorganisms in the BD3526 group were not wholly increased. Therefore, it could be postulated that both *A. muciniphila* and SCFAs-producing microorganisms are important in the regulation of blood glucose and behaved differently.

*Akkermansia muciniphila* is an anaerobic bacterium attached to the intestinal mucosa (Derrien et al., 2004, 2008; Collado et al., 2007; van Passel et al., 2011), and a lower level of *A. muciniphila* has been reported in obesity and T2DM subjects compared with normal ones. In addition, *A. muciniphila* can also increase the patient’s response to drugs by increasing the recruitment of CCR9^+^ CXCR3^+^ CD4^+^ T lymphocytes during the immunotherapy of tumor patients with PD-1/PD-L1 and increase the progression-free survival (PFS) of patients after treatment (Routy et al., 2018). Although the molecular mechanism by which *A. muciniphila* ameliorates diabetes/obesity is not fully understood, it is widely believed that this bacterium can play a positive role in health promotion by increasing the integrity of the intestinal mucosa (Derrien et al., 2004, 2008; Collado et al., 2007).

In T2DM, although the direct cause of the syndrome is insufficient for insulin secreted by pancreatic β-cells, the intrinsic cause is the inflammatory response (Cuman et al., 2001; Naguib et al., 2004). The chronically low grade of inflammatory responses not only causes pancreatic β-cells to be attacked but also reduces the liver and muscle sensitivity to insulin, which results in insulin resistance (Cerasi, 1995; Kahn, 1998; Bonner-Weir, 2000; Kahn et al., 2006). Even less optimistic is that the inflammatory response not only causes diabetes but also shows correlation between T2DM, insulin resistance, cardiovascular disease and obesity (Donath, 2013). Therefore, reducing inflammation has been an important strategy for diabetes treatment. In our work, we found that the BD3526 strain fermentation products could lower the expression of intestinal mucosal inflammatory factors in GK rats and the level of HaB1c and blood glucose.

In this work, we used the BD3526 strain isolated from Tibetan yak milk to ferment skim milk (Hang et al., 2016). Although our understanding of this strain is not comprehensive, it has already demonstrated some potential in health promotion, e.g., secreting antimicrobial agents and levan, which is a recognized dietary fiber (Xu et al., 2016). Since these fermentation products could alleviate the symptoms of T2DM and selectively stimulate the propagation of *A. muciniphila* in the intestine, we believe that the biological function of *P. bovis* sp. nov. BD3526 might not be restricted to improving diabetes. As only the metabolites of *P. bovis* sp. nov. BD3526 in skim milk are employed in this work, the effect of the bacterium itself on the intestinal tract is still unknown and worthy of further investigation.

## Author Contributions

ZQ, JH, ZW, and ZL conceived and designed the experiments. JH, HF, MY, CG, JW, and CY performed the experiments. ZQ, HZ, CG, and CY analyzed the data. ZQ and JH wrote the manuscript. All the authors read and approved the final manuscript.

## Conflict of Interest Statement

ZQ, JH, HF, CG, JW, CY, ZL, and ZW were employed by Bright Dairy & Food Co., Ltd. The remaining authors declare that the research was conducted in the absence of any commercial or financial relationships that could be construed as a potential conflict of interest.

## Footnotes

1 http://drive5.com/uparse/

2 http://www.mothur.org/wiki/Schloss_SOP#Alpha_diversity

3 http://networkx.github.io
