# Supplementary material for: Fermentation Products of Paenibacillus bovis sp. nov. BD3526 Alleviates the Symptoms of Type 2 Diabetes Mellitus in GK Rats
Source: Front Microbiol. 2019 Jan 9;9:3292. doi: 10.3389/fmicb.2018.03292 (PMC6333654; doi:10.3389/fmicb.2018.03292)
Supplement: Supplementary file 1 [file Data_Sheet_1.docx]

Supplementary Material

Metabolites of *Paenibacillus bovis* sp.nov. BD3526 alleviates symptoms of type 2 diabetes mellitus in GK rats

Zhenyi Qiao, Zhengjun Wu*

*** Correspondence:** Corresponding Author: [wuzhengjun@brightdairy.com](mailto:wuzhengjun@brightdairy.com)

# Supplementary Figures and Tables

## Supplementary Figures


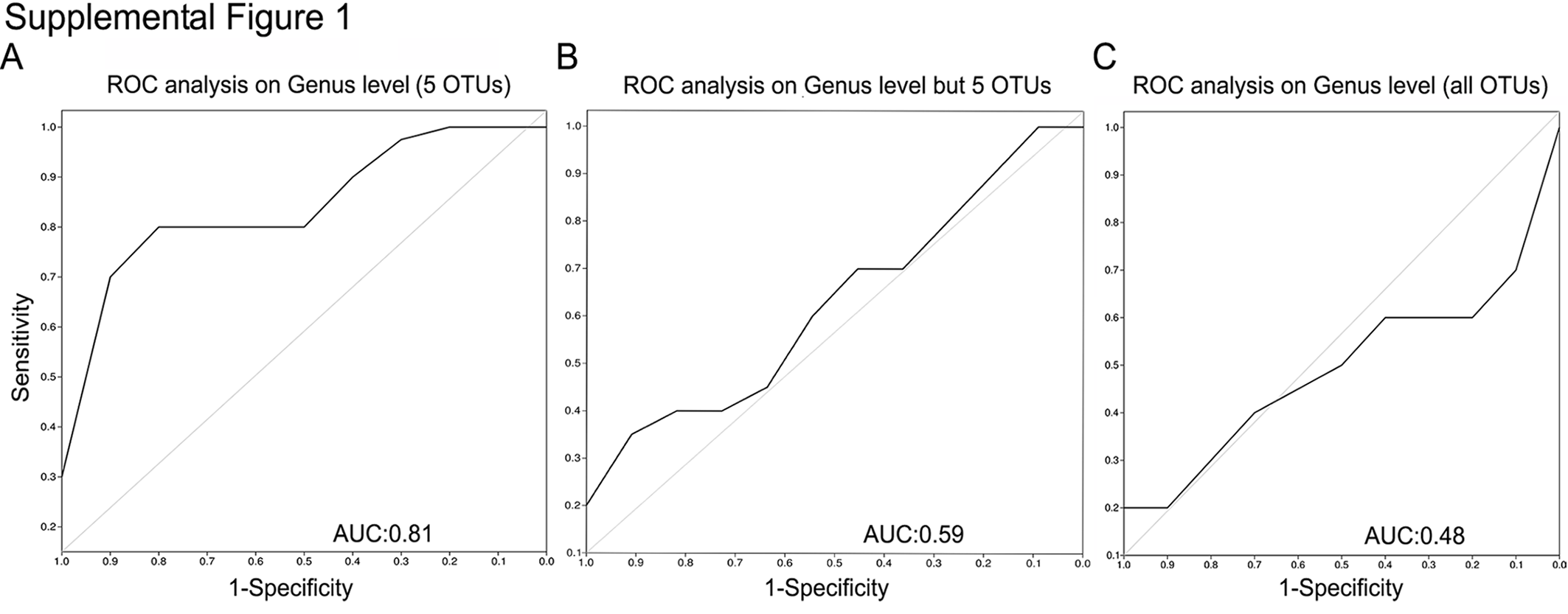


**Supplementary Figure 1: ROC curve of 5 specific gut microbes in the BD3526 group.**

**A.** ROC analysis on genus level (5 OTUs). **B.** ROC analysis on genus level but 5 OTUs. **C.** ROC analysis on genus level (All OTUs).

**
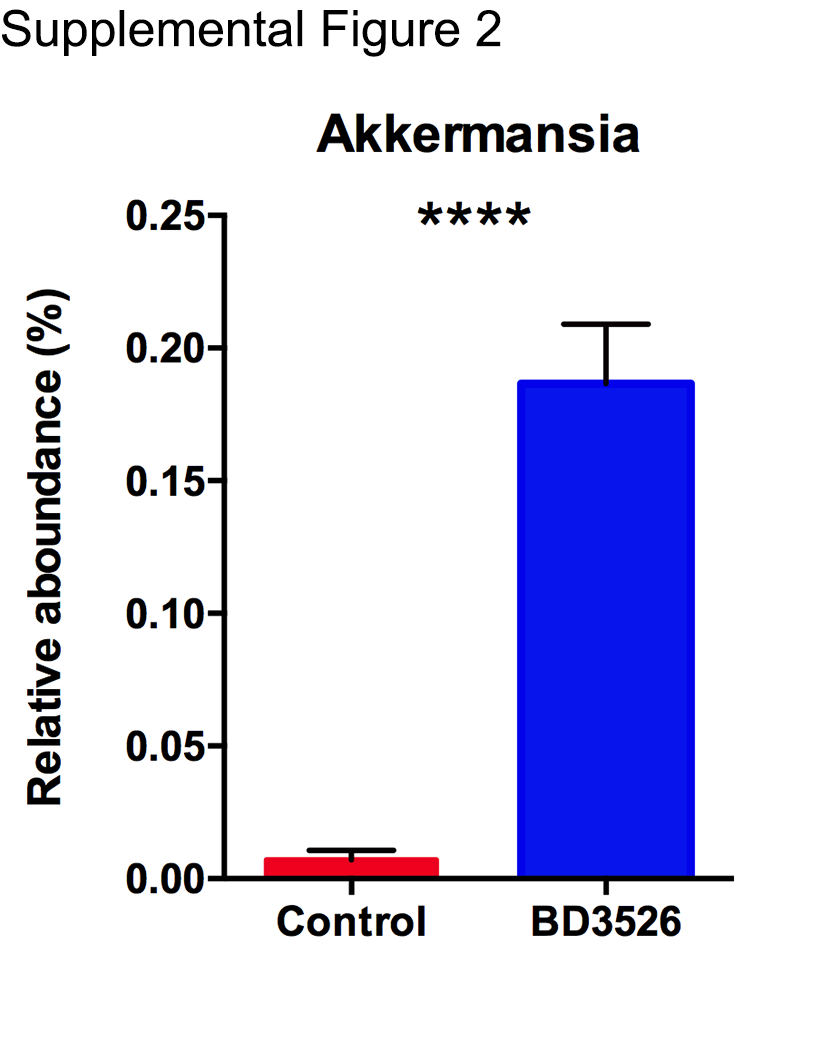
**

**Supplementary Figure 2: Relative abundance of *Akkermansia* in vitro experiment. (**** P-value <0.0001, mean ± SEM).**


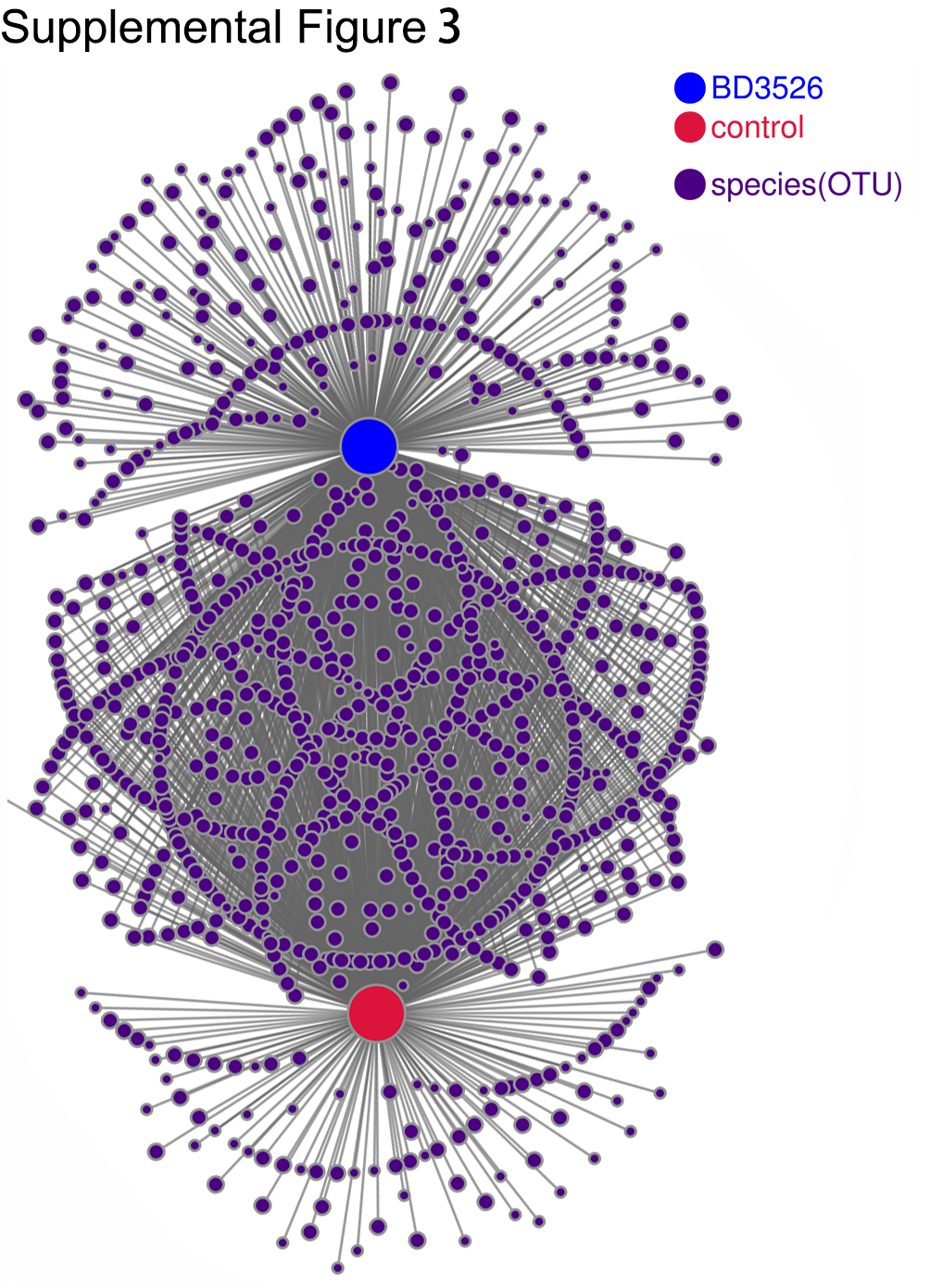


**Supplementary Figure 3: Strain specificity analysis of the BD3526 group and the control group.**

The blue cycle represents the BD3526 group and the red cycle represents the control group. The purple cycles represent the species that was identified in this experiment. The purple cycles of which they are combined in both BD3526 group and control group by lines are regarded as common species in both two groups. And the purple cycles combined by one line represent existing in only one group. The longer the lines, the higher the importance.

**1.2 Supplementary Tables**

**Supplementary Table 1: Metagenomeseq analysis of differential gut microbial list.**

| Taxa | fidherP | fisherAdjP | pvalues | adjPvalues |
| --- | --- | --- | --- | --- |
| Akkermansia | 0.003095975 | 0.492260062 | 0.00141646 | 0.185573239 |
| Alkaliphilus | 0.03250774 | 0.738390093 | 0.00357775 | 0.185573239 |
| Sulfurimonas | 0.03250774 | 0.738390093 | 0.006801951 | 0.185573239 |
| Amphritea | 0.03250774 | 0.738390093 | 0.006933818 | 0.185573239 |
| Ruminococcaceae_NK4A214 | 0.086687307 | 0.810781278 | 0.006998041 | 0.185573239 |
| Ruminiclostridium_1 | 0.086687307 | 0.810781278 | 0.007002764 | 0.185573239 |
| Prevotella_9 | 1 | 1 | 0.01116906 | 0.209298478 |
| Photobacterium | 0.086687307 | 0.810781278 | 0.012332431 | 0.209298478 |
| Lactococcus | 0.473684211 | 1 | 0.013582076 | 0.209298478 |
| Arenibacter | 0.03250774 | 0.738390093 | 0.01401634 | 0.209298478 |
| Anaerolineaceae | 0.03250774 | 0.738390093 | 0.015159438 | 0.209298478 |
| Flavobacteriaceae | 0.03250774 | 0.738390093 | 0.015796112 | 0.209298478 |
| Sulfurovum | 0.140866873 | 1 | 0.018872888 | 0.224579468 |
| Enterobacteriaceae | 1 | 1 | 0.019945994 | 0.224579468 |
| Colwellia | 0.086687307 | 0.810781278 | 0.021186742 | 0.224579468 |
| Clostridium_sensu | 0.086687307 | 0.810781278 | 0.027476689 | 0.273049594 |
| Peptococcaceae | 1 | 1 | 0.031754608 | 0.280501224 |
| Magnetovibrio | 0.210526316 | 1 | 0.033338396 | 0.280501224 |
| Thiotrichaceae | 0.210526316 | 1 | 0.033519014 | 0.280501224 |
| Lactobacillus | 1 | 1 | 0.040007918 | 0.310568236 |
| Rhodobacteraceae | 0.210526316 | 1 | 0.042815971 | 0.310568236 |
| Lachnospiraceae_ND3007 | 0.473684211 | 1 | 0.042971706 | 0.310568236 |
| Coprococcus_2 | 0.649916647 | 1 | 0.049864312 | 0.32747532 |

**Supplementary Table 2: Random forest distribution analysis of differential gut microbial list.**

| Feature | Importance |
| --- | --- |
| Akkermansia | 6.02345 |
| Candidatus_Saccharimonas | 5.26068 |
| Ruminiclostridium_9 | 3.63136 |
| norank_f__Anaerolineaceae | 3.36316 |
| Prevotella_9 | 3.05411 |
| Lactococcus | 2.93982 |
| Alistipes | 2.63889 |
| norank_f__Peptococcaceae | 2.48648 |
| Tyzzerella | 2.46813 |
| Amphritea | 2.46769 |
| Bacteroides | 2.41203 |
| Sulfurimonas | 2.40128 |
| unclassified_f__Enterobacteriaceae | 2.21544 |
| norank_f__Bacteroidales_S24_7_group | 2.188 |
| unclassified_c__Alphaproteobacteria | 2.16165 |
| Christensenellaceae_R_7_group | 2.1089 |
| Alkaliphilus | 2.07727 |
| Ruminiclostridium_1 | 2.05314 |
| Allobaculum | 2.00225 |
| Coprococcus_1 | 1.94471 |
| unclassified_f__Veillonellaceae | 1.92846 |
| Lactobacillus | 1.91381 |
| Alloprevotella | 1.8831 |
| unclassified_f__Flavobacteriaceae | 1.85926 |
| Ruminiclostridium | 1.85298 |
| Jeotgalicoccus | 1.74847 |
| Pseudomonas | 1.68271 |
| Prevotellaceae_NK3B31_group | 1.604 |
| Streptococcus | 1.53616 |
| Nitrospira | 1.4164 |
| unclassified_f__Rhodobacteraceae | 1.41631 |
| norank_o__NB1_n | 1.41621 |
| Psychrobacter | 1.40275 |
| norank_f__Thiotrichaceae | 1.38943 |
| unclassified_o__Bacteroidales | 1.38617 |
| Turicibacter | 1.34903 |
| unclassified_f__Peptostreptococcaceae | 1.28175 |
| Treponema_2 | 1.16418 |
| norank_f__Coriobacteriaceae | 1.16091 |
| Ruminiclostridium_5 | 1.14282 |
| Blautia | 1.08011 |
| Thioalkalispira | 1.001 |
| norank_f__Sva1033 | 1.001 |
| Mucispirillum | 1.001 |
| Photobacterium | 1.001 |
| Faecalibacterium | 1.001 |
| Devosia | 1.001 |
| unclassified_p__Bacteroidetes | 1.001 |
| Lachnospiraceae_NK4B4_group | 1.001 |
| norank_o__Subgroup_6 | 1.001 |
| Dielma | 1.001 |
| Colwellia | 1.001 |
| Helicobacter | 0.9186 |
| Ruminococcaceae_UCG_010 | 0.89735 |
| Lachnospiraceae_UCG_010 | 0.86142 |
| Ruminococcaceae_UCG_009 | 0.84561 |
| unclassified_f__Coriobacteriaceae | 0.79138 |
| Ruminococcus_1 | 0.74524 |
| Lachnospiraceae_FCS020_group | 0.7359 |
| Sulfurovum | 0.70449 |
| Parasutterella | 0.70224 |
| Prevotellaceae_Ga6A1_group | 0.70134 |
| norank_f__Erysipelotrichaceae | 0.69291 |
| Gemella | 0.64704 |
| Arenibacter | 0.63355 |
| Rothia | 0.61208 |
| norank_f__Lachnospiraceae | 0.52035 |
| Rikenellaceae_RC9_gut_group | 0.51848 |
| Caldicoprobacter | 0.50091 |
| Ruminococcus_gauvreauii_group | 0.47358 |
| Family_XIII_UCG_001 | 0.4696 |
| unclassified_f__Family_XIII | 0.43829 |
| Barnesiella | 0.43416 |
| Coprococcus_2 | 0.43115 |
| norank_f__Christensenellaceae | 0.3218 |
| unclassified_p__Firmicutes | 0.28136 |
| Peptococcus | 0.24799 |
| Anaerovorax | 0.22165 |
| Ruminococcaceae_UCG_008 | 0.20001 |
| unclassified_f__Prevotellaceae | 0.10595 |
| Ruminococcaceae_UCG_014 | 0.08553 |
| Sulfitobacter | 0 |
| Delftia | 0 |
| norank_o__Subgroup_21 | 0 |
| Papillibacter | 0 |
| Aminobacter | 0 |
| norank_o__Sh765B_TzT_29 | 0 |
| Erysipelotrichaceae_UCG_003 | 0 |
| Faecalitalea | 0 |
| Collinsella | 0 |
| norank_c__TK10 | 0 |
| norank_f__NS9_marine_group | 0 |
| Hydrogenoanaerobacterium | 0 |
| Anaerostipes | 0 |
| norank_c__MACA_EFT26 | 0 |
| Acidibacter | 0 |
| norank_f__Sva0996_marine_group | 0 |
| Fusibacter | 0 |
| Acetatifactor | 0 |
| norank_f__JTB255_marine_benthic_group | 0 |
| Desulfobulbus | 0 |
| norank_c__S085 | 0 |
| Candidatus_Arthromitus | 0 |
| Victivallis | 0 |
| Thiohalophilus | 0 |
| Acetitomaculum | 0 |
| Lachnospiraceae_UCG_001 | -0.02199 |
| Ruminococcaceae_UCG_013 | -0.1344 |
| Oscillibacter | -0.17566 |
| Tyzzerella_3 | -0.18572 |
| Marvinbryantia | -0.23669 |
| unclassified_f__Erysipelotrichaceae | -0.25474 |
| Corynebacterium_1 | -0.2807 |
| Elusimicrobium | -0.31924 |
| Desulfovibrio | -0.35156 |
| norank_f__Clostridiales_vadinBB60_group | -0.40038 |
| Clostridium_sensu_stricto_13 | -0.41183 |
| Lachnospiraceae_NK4A136_group | -0.43021 |
| unclassified_o__Clostridiales | -0.4553 |
| Family_XIII_AD3011_group | -0.51625 |
| Eubacterium_brachy_group | -0.52704 |
| Butyricimonas | -0.52877 |
| Lachnospiraceae_ND3007_group | -0.53587 |
| Roseburia | -0.5589 |
| Anaerotruncus | -0.56474 |
| Candidatus_Stoquefichus | -0.66256 |
| Catabacter | -0.70193 |
| Ruminococcaceae_NK4A214_group | -0.74235 |
| Butyrivibrio | -0.80519 |
| Lachnospiraceae_UCG_006 | -0.81135 |
| Ruminococcus_2 | -0.81739 |
| Lachnospiraceae_UCG_005 | -0.82659 |
| Ruminococcaceae_UCG_005 | -0.88647 |
| unclassified_k__norank | -0.93525 |
| Eubacterium_ventriosum_group | -0.95952 |
| Escherichia_Shigella | -0.98585 |
| Magnetovibrio | -1.001 |
| Defluviitaleaceae_UCG_011 | -1.001 |
| norank_o__Opitutae_vadinHA64 | -1.001 |
| Actinomyces | -1.001 |
| Pasteurella | -1.001 |
| Eubacterium_nodatum_group | -1.001 |
| Enterorhabdus | -1.08115 |
| Intestinimonas | -1.14325 |
| Anaeroplasma | -1.2376 |
| norank_o__Gastranaerophilales | -1.28963 |
| Coriobacteriaceae_UCG_002 | -1.29335 |
| Rikenella | -1.34406 |
| Ruminiclostridium_6 | -1.40323 |
| Adlercreutzia | -1.41895 |
| Erysipelatoclostridium | -1.61656 |
| Eubacterium_coprostanoligenes_group | -1.71301 |
| unclassified_f__Ruminococcaceae | -1.8102 |
| Parabacteroides | -1.81699 |
| norank_f__Ruminococcaceae | -1.8511 |
| Aerococcus | -1.91128 |
| Thalassospira | -2.06825 |
| unclassified_f__Lachnospiraceae | -2.42894 |
| norank_o__Mollicutes_RF9 | -2.6436 |
